# An Unusual Stress Metabolite from a Hydrothermal Vent Fungus *Aspergillus* sp. WU 243 Induced by Cobalt

**DOI:** 10.3390/molecules21010105

**Published:** 2016-01-16

**Authors:** Chihong Ding, Xiaodan Wu, Bibi Nazia Auckloo, Chen-Tung Arthur Chen, Ying Ye, Kuiwu Wang, Bin Wu

**Affiliations:** 1 Ocean College, Zhejiang University, Hangzhou 310058, China; dingchihong@163.com (C.D.); wxd_zju@163.com (X.W.); naz22ia@hotmail.com (B.N.A.); ctchen@faculty.nsysu.edu.tw (C.-T.A.C.); gsyeying@zju.edu.cn (Y.Y.); 2 Institute of Marine Geology and Chemistry, National Sun Yat-sen University, Kaohsiung 80424, Taiwan; 3 Department of Applied Chemistry, Zhejiang Gongshang University, Hangzhou 310058, China; wkwnpc@zjgsu.edu.cn

**Keywords:** marine fungi, metal-stress, hydrothermal vent, marine natural products

## Abstract

A novel hybrid polyketide-terpenoid, aspergstressin (**1**), possessing a unique fused polycyclic structure, was induced from culture broth of strain *Aspergillus* sp. WU 243 by cobalt ion stimulation. The strain was isolated from the digestive gland of *Xenograpsus testudinatus*, a unique type of crab which dwells in the Kueishantao hydrothermal vents off Taiwan. The chemical structure and relative configuration of the stress metabolite were established by spectroscopic means. *Aspergillus* sp. WU 243 produced aspergstressin (**1**) only under cobalt stressed culture conditions. The results show that stress-driven discovery of new natural products from hydrothermal vent fungi is an effective strategy to unveil the untapped reservoir of small molecules from species found in the hydrothermal vent environment.

## 1. Introduction

Marine hydrothermal vent microorganisms adapt and respond rapidly to changes in the availability and concentrations of metals within their harsh and dynamic environment [1,2]. Marine organisms, living in a biologically competitive and stressful habitat, are of great interest as new promising sources of biologically active products [3,4,5]. Contrary to the perception that metals hinder secondary metabolite production, recent studies have showed that metals can induce or enhance the synthesis of possibly potent and medically relevant metabolites [5,6,7]. Arousing sleeping genes or producing structures with stereochemical features which facilitate metal complexation and their transportation in biological systems may account for the mechanisms behind the metal-induced metabolite phenomenon [7,8].

In this study, the metal-stress method was applied on a marine fungus strain isolated from the digestive gland of *Xenograpsus testudinatus*, which was collected from a Kueishantao Island hydrothermal vent in Taiwan. The strain was identified as *Aspergillus* sp. by comparing 18S ribosomal RNA genes with database information. As a result, a new hybrid polyketide-terpenoid named aspergstressin (**1**) induced by cobalt ion was identified as the new stress metabolite.

## 2. Results and Discussion

### 2.1. Identification of Strain WU 243

**Figure 1:**
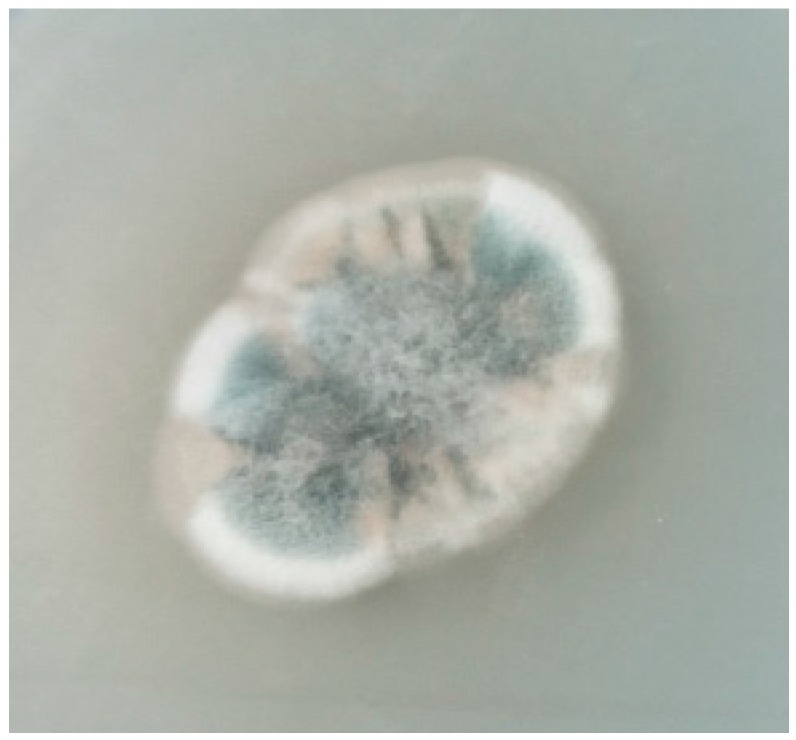
*Aspergillus* sp. strain WU 243, agar colony on PDA (PD agar) medium after 7 days of incubation at 24 °C.

Strain WU 243 were isolated from the digestive gland of *Xenograpsus testudinatus*, a unique type of arthropod which inhabits in the Kueishantao hydrothermal vents off northeast Taiwan. The strain was cultured on PD agar (PDA) medium and grew as white mycelium covered with deep green spores (Figure 1). According to the morphological criteria for identification, *Aspergillus* and *Penicillium* were both potential candidates.

The sequences of the 18S rRNA genes for strain WU 243 comprised 593 nucleotides. The highest sequence similarity (100%) was observed for the strains *Aspergillus* sp. BMP3039 18S ribosomal RNA gene, internal transcribed spacer 1, 5.8S ribosomal RNA gene, internal transcribed spacer 2, and 28S ribosomal RNA gene region and *Aspergillus versicolor* strain LTBF 011-1 18S ribosomal RNA gene, partial sequence; internal transcribed spacer 1, 5.8S ribosomal RNA gene, and internal transcribed spacer 2, complete sequence; and 28S ribosomal RNA gene, partial sequence. From the above results, strain WU 243 was classified as an *Aspergillus* sp.

### 2.2. Metabolic Profiles of the Strain WU 243 after Metal-Stress Application and Optimization of Stress Fermentation

**Figure 2:**
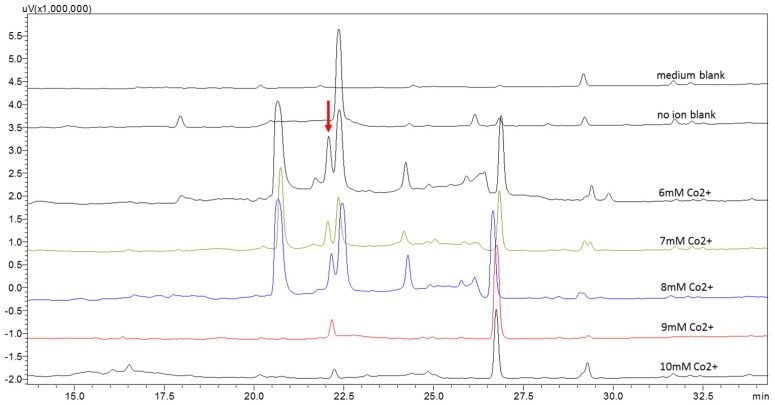
Metabolic profile of strain WU 243 by HPLC analysis: blank control and six sets of different ion concentrations.

**Figure 3:**
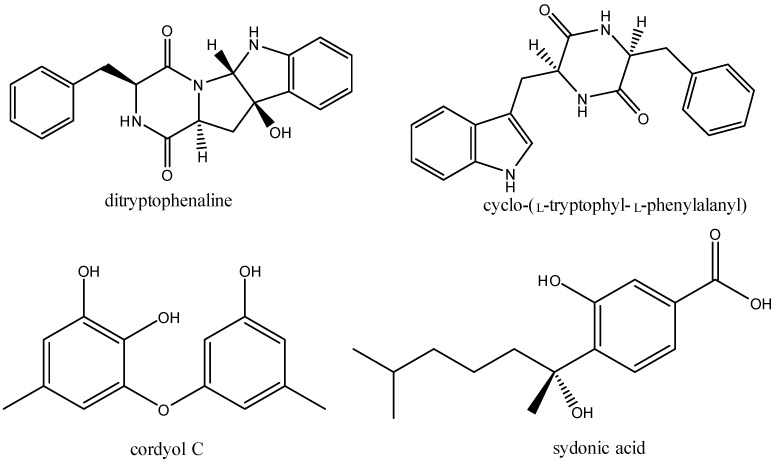
Structures of known compounds.

Cultivation experiments of strain WU 243 were carried out in potato dextrose broth (PDB) media under static conditions. The strain was tested for its susceptibility to cadmium, zinc, cobalt, and nickel, and results showed that only cobalt induced the production of stress metabolites. The change in metabolic pattern after cobalt ion induction was verified by setting up one medium without fungus as a blank control, one medium without ions and six sets of media with different ion concentrations (6 mM to 10 mM). Mycelium and culture broth were separated by a gauze filter and the culture broth was extracted with ethyl acetate. Results showed that a new peak (compound **1**) appeared, which was almost invisible in the non-metal treated fungal extract. Among the different concentrations, the concentration of 6 mM cobalt was observed as the most effective (Figure 2). More interestingly, the production of the normal main product appearing at 22.5 min, which was purified and identified as the known compound ditryptophenaline [9] (Figure 3), was drastically reduced under metal stress. When the concentration of cobalt reached 9 mM, the main product at 22.5 min almost ceased to secrete, and stress metabolites were overwhelmingly the major products in the extract. Three new peaks (retention times: 20.5, 24.25 and 26.75 min, respectively) in addition to compound **1** were found in traces of the cobalt-containing cultures. These compounds were purified by preparative HPLC and identified as the known compounds cyclo-(l-tryptophyl-l-phenylalanyl) [10], cordyol C [11] and sydonic acid [12] (Figure 3). The NMR data and assignments of the known compounds are available in the Supplementary Materials.

The metal-stress experiment was first carried out by setting four cobalt ion concentrations of 0, 5, 10, and 20 mM, respectively. The strain was cultured for 14 days at 24 °C under static conditions. It was observed that the growth of the strain was inhibited in 20 mM cobalt ion medium. The mycelium was removed and culture broth was extracted with ethyl acetate in equal volume. The HPLC profile of each extract showed the fungal metabolic response to ion stimulation when the cobalt ion concentration was up to 5 mM. When the cobalt ion concentration was 10 mM, few secondary metabolites were detected.

**Figure 4:**
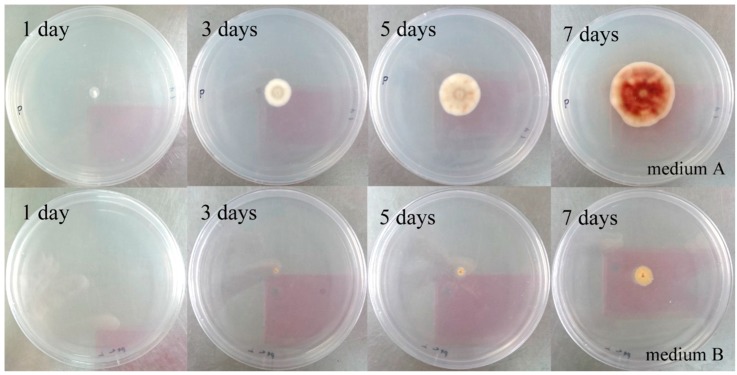
Fungal colonies in medium A (agar plates with PDA) and medium B (agar platess with PDA and 5 mM CoCl_2_) after 1, 3, 5 and 7 days of incubation.

In order to improve the stress metabolite yield, the conditions for stress fermentation have been optimized. Since cobalt ions are toxic to fungi, the growth rate was significantly inhibited when the cobalt ion concentration reached 5 mM in PDA medium (Figure 4). However, according to the primary experimental results, stress products could be detected only when the cobalt ion concentration was up to 5 mM. Therefore, enhancing the production of the stress metabolites in the range of ion concentrations with good growth rate was important. The fungus was treated with five different ion concentrations from 6 mM to 10 mM. The HPLC profile of each extract revealed the peak area of stress compound **1** was maximized at 6 mM cobalt ion concentration and it decreased with increasing ion concentrations. Hence, 6 mM cobalt ion concentration was selected for large scale stress fermentations.

In order to accelerate the growth of the fungus and the accumulation of metabolites, the fermentation temperature was raised to 28 °C and the duration of cultivation was prolonged to 18 days. Twenty L of zymotic fluid were placed in flasks of two sizes, 500 mL and 5 L, under static conditions.

### 2.3. Structural Elucidation

**Figure 5:**
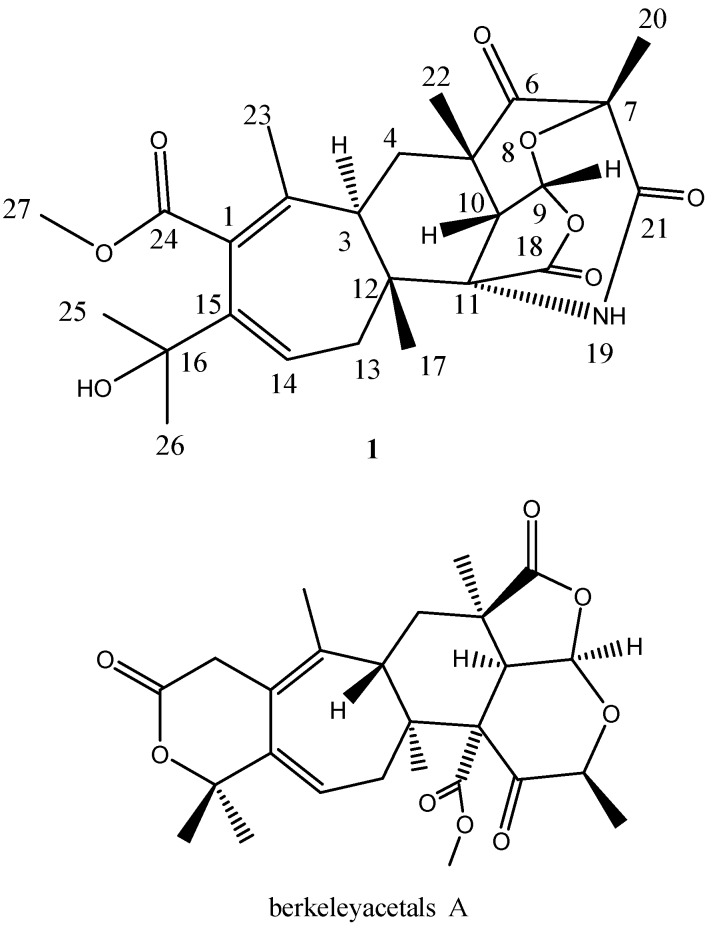
Structures of compound **1** and berkeleyacetal A [13].

After large scale fermentation, the culture broth was collected and extracted with ethyl acetate. The extracts of the broth of strain WU 243 were then subjected to column chromatography and preparative HPLC to obtain purified compound **1** (Figure 5).

**Figure 6:**
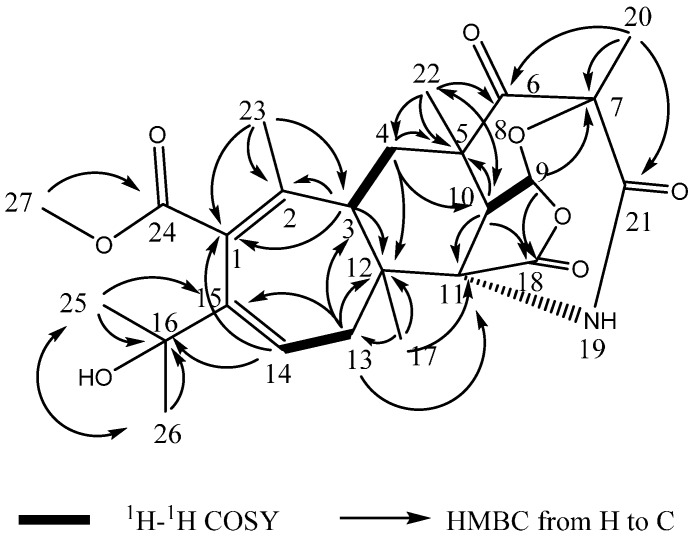
Key ^1^H-^1^H COSY and HMBC correlations of compound **1**.

Compound **1** was isolated as a white powder. The molecular formula C_25_H_31_NO_8_ was determined by analysis of the HR-TOF-MS data. The formula was supported by the ^13^C-NMR data, which indicated 11 degrees of unsaturation. The NMR data (Table 1) of compound **1** were similar to those of the berkeleyacetals analogues [13]. These molecules contain two distinct domains, a tricyclic hydrocarbon and a δ-lactone ring, connected by a seven member ring [13]. The skeleton of fused polycyclic system contains a seven membered ring, a six membered ring, a five membered lactone ring and six membered ketonic ether ring. The 25 carbon signals comprised seven methyls (δ_C_ 16.5, 26.4, 26.8, 27.8, 30.7, 32.3 and 54.7), two methylenes (δ_C_ 32.4, 40.3) and three methines (δ_C_ 44.8, 45.2 and 100.0). A comparison between the DEPT 135 and ^13^C-NMR spectra revealed five quaternary carbons (δ_C_ 48.0, 51.7, 56.6, 81.8 and 85.6), two sets of double bonds (127.9 and 131.5, 139.8 and 140.9) and four carbonyls (δ_C_ 211.2, 173.2, 174.3 and 173.2). The ^13^C-NMR spectrum of **1** showed the presence of 14 signals for the hydrocarbon domain, including a tricyclic basic skeleton, with the remaining 11 resonances corresponding to a cyclohepta-1,3-diene with a dimethyl carbinol moiety and a methoxycarbonyl group. When compared to the NMR data of berkeleyacetal A [13], isolated from the fungus *Penicillium* sp. growing in Berkeley Pit Lake, compound **1** showed similar chemical shifts of the main fused polycyclic skeleton and the seven membered ring unit. However, 1D and 2D NMR analysis revealed that the substructure of tricyclic hydrocarbon and the units attached on it were less similar to berkeleyacetal A. In the ^1^H-^1^H COSY spectrum of compound **1** (Figure 6), the olefin proton at δ_H_ 6.39 (dd, *J* = 8.0, 5.2 Hz, H-14) was coupled with two methylene protons at δ_H_ 2.51 (dd, *J* = 13.2, 8.0 Hz, H-13α) and 1.78 (dd, *J* = 13.2, 5.2 Hz, H-13β), and the methine protons at δ_H_ 2.06 (dd, *J* = 13.5, 2.1 Hz, H-3) were coupled with two methylene protons at δ_H_ 1.50 (t, *J* = 13.5 Hz, H-4α) and 2.39 (dd, *J* = 13.6, 2.6 Hz, H-4β). The methine proton at δ_H_ 6.09 (d, *J* = 7.1 Hz, H-9) exhibited cross peaks with methine proton at δ_H_ 3.27 (d, *J* = 7.1 Hz, H-10) in the COSY spectrum of **1**. The sequence and linkages of four ring system of H-3/H2-4/H-9/H-10/H2-13/H-14 was deduced from the above ^1^H-^1^H COSY analyses (Figure 6). HMBC correlation from Me-20 to carbonyl C-21 indicated that the carbonyl located at C-21 and the formation of the amide with the help of the molecular formula information (Figure 6). HMBC cross peaks of Me-20/C-6, Me-20/C-7 and Me-20/C-21 positioned a methyl at C-7. HMBC cross peaks of Me-22/C-4, Me-22/C-5, Me-22/C-6 and Me-22/C-10 assigned a methyl group at C-5 and positioned a ketone group at C-6. HMBC correlations of H-9/C-5, H-9/C-7, H-9/C-10, H-9/C-18, H-10/C-5, H-10/C-6, H-10/C-9, H-10/C-11, H-10/C-18 and H-10/C-22, together with the HMBC cross peaks of Me-22/C-10 and Me-22/C-6 revealed that a rearranged polyketide fused ring structure was formed. The long range correlations of Me-17/C-3, H-4/C-12 and Me-17/C-11 connected the fused three rings with the seven membered olefinic ring. In the ^1^H-NMR spectrum of **1**, seven methyl groups displayed six singlet signals (H-20 and H-25 were overlapped as one peak), one of which was assigned at olefinic quaternary C-2 from the observation of HMBC cross peaks of Me-23/C-1, Me-23/C-2 and Me-23/C-3. The long range correlation from the proton signal at δ_H_ 3.67 (s, Me-27) to the ester carbonyl signals at δ_C_ 137.2 (C-24) positioned the OMe attached at carbonyl 24. The HMBC correlations of H-14/C-16, H-25/C-15, H-13/C-15 and H-25/C-26 revealed that the lactone ring at the carbon backbone of berkeleyacetal A [13] was opened and rearranged, and a dimethyl carbinol group was attached at C-15. The quaternary carbon signal at δ_C_ 51.7 was attributed to C-11 from the analysis of long range correlations from the proton signals at δ_H_ 1.04 (s, H-17) to quaternary C-11. The proton signals at δ_H_ 3.28 (s, H-10) and 1.77 (s, H-13) also showed diagnostic HMBC correlations with δ_C_ 51.7. Considering the linkage with C-10, C-12 and C-18 and the chemical shift of quaternary carbon C-11, the amide bridge was formed between C-7 and C-11.

The main differences between the structures of **1** and the berkeleyacetals are the absence of the lactone ring and rearrangement of the bicyclic decahydrofuro[4,3,2-ij]isochromene moiety. In the structure of **1**, the lactone ring at C-1 and C-15 was opened, with a dimethyl carbinol assigned at C-15 and a methoxycarbonyl group at C-1. The HMBC correlations between H-3 and C-2, C-3, C-4 and the long range correlations from Me-17 to C-11, C-12, and C-13 confirmed the linkage between the seven membered ring and three fused rings. By comparing NMR data with those of berkeleyacetal A in the literature [13], the three fused ring substructure was similar to that in berkeleyacetal A, however, the positions of the tetrahydropyran ring and tetrahydrofuran ring in the fused three ring substructure was rearranged as drawn in Figure 5. Diagnostic carbon signals of Me-22 (δ_C_ 32.3), and the two methine proton signals at H-9 (δ_H_ 6.09, d, *J* = 7.1 Hz) and H-10 (δ_H_ 3.28, d, *J* = 7.1 Hz) appeared in the ^1^H- and ^13^C-NMR spectra of **1**. The HMBC cross peak of H-10/carbonyl C-6 and the cross peak of H-9/C-18 confirmed that the three fused ring substructure was rearranged. Detailed two-dimensional NMR analysis permitted the assignment of the polycyclic hydrocarbon unit. Thus, the planar structure of **1** was elucidated as shown in Figure 5.

**Table 1:** NMR data for compound **1** (600 MHz, CD_3_OD).

Position	δ_C_^a,b^, Mult.	δ_H_^c^, Mult. (*J* in Hz)	HMBC	NOESY
1	127.9			
2	139.8			
3	44.8, CH	2.07, dd (13.5, 2.1)	C-1/C-2/C-12/C-17/C-23	H-4β
4β	32.4, CH_2_	1.49, t (13.5)	C-3/C-5/C-6/C-12/C-22	H-4β/H-23/H-17/H-22
4α		2.39, dd (13.6, 2.6)	C-5/C-6/C-10/C-12	H-4α/H-22/H-3/H-23
5	48.0			
6	211.2			
7	81.8			
8	O			
9	100.0, CH	6.10,d (7.1)	C-5/C-7/C-10/C-18	H-10
10	45.2, CH	3.27, d (7.1)	C-5/C-6/C-9/C-11/C-18/C-22	H-9/H-17/H-22
11	51.7			
12	56.6			
13α	40.3, CH_2_	2.51, dd (13.2, 8.0)	C-3/C-11/C-12/C-14/C-15	H-13β/H-14
13β		1.78, dd (13.2, 5.2)	C-11/C-12/C-14/C-15/C-17	H-13α/H-17
14	131.5, CH	6.40,dd (8.0, 5.2)	C-1/C-12/C-13/C-16	H-25/H-13α
15	140.9			
16	85.6			
17	26.4, CH_3_	1.04, s	C-3/C-11/C-12/C-13/C-14/C-18	H-10/H-13β/H-4α
18	174.3			
19	N			
20	26.8, CH_3_	1.56, s	C-6/C-7/C-21	H-22
21	173.2			
22	32.3, CH_3_	1.40, s	C-4/C-5/C-6/C-10	H-10/H-20/H-4α/H-4β
23	16.5, CH_3_	1.83, s	C-1/C-2/C-3	H-4α/H-4β
24	173.2			
25	27.8, CH_3_	1.56,s	C-15/C-16/C-26	H-14/H-26
26	30.7, CH_3_	1.39, s	C-15/C-16/C-25	H-25
27	54.7, CH_3_	3.67, s	C-24	

^a^ Recorded at 150 MHz; ^b^ Multiplicities inferred from DEPT and HSQC experiments; ^c^ Recorded at 600 MHz.

**Figure 7:**
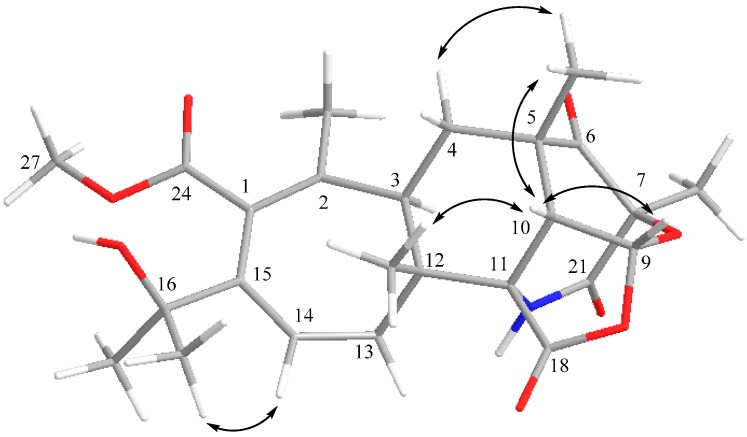
Key NOESY correlations of compound **1**.

The relative configuration of compound **1** was determined by NOESY experiments. NOESY cross peaks of H-14/H-25, H-17/H-10 and H-10/H-22 revealed a cyclohexyl chair ring with a β-oriented Me-17 at C-12 and a fused six membered pyrone boat ring with a β-oriented Me-20 at C-7 and a β-oriented Me-22 at C-5 (Figure 7). Since no NOESY correlation between H-3 and Me-17 was observed, the cyclohepta-1,3-diene ring and cyclohexane ring were *trans*-fused. H-10 showed NOESY cross peaks with H-9, Me-17 and Me-22, indicating that pyran ring and tetrahydrofuran ring were *cis*-fused, and H-9 and H-10 were β-oriented. Thus, compound **1** was identified as a new compound and named aspergstressin.

### 2.4. Proposed Biosynthetic Pathway

**Scheme 1:**
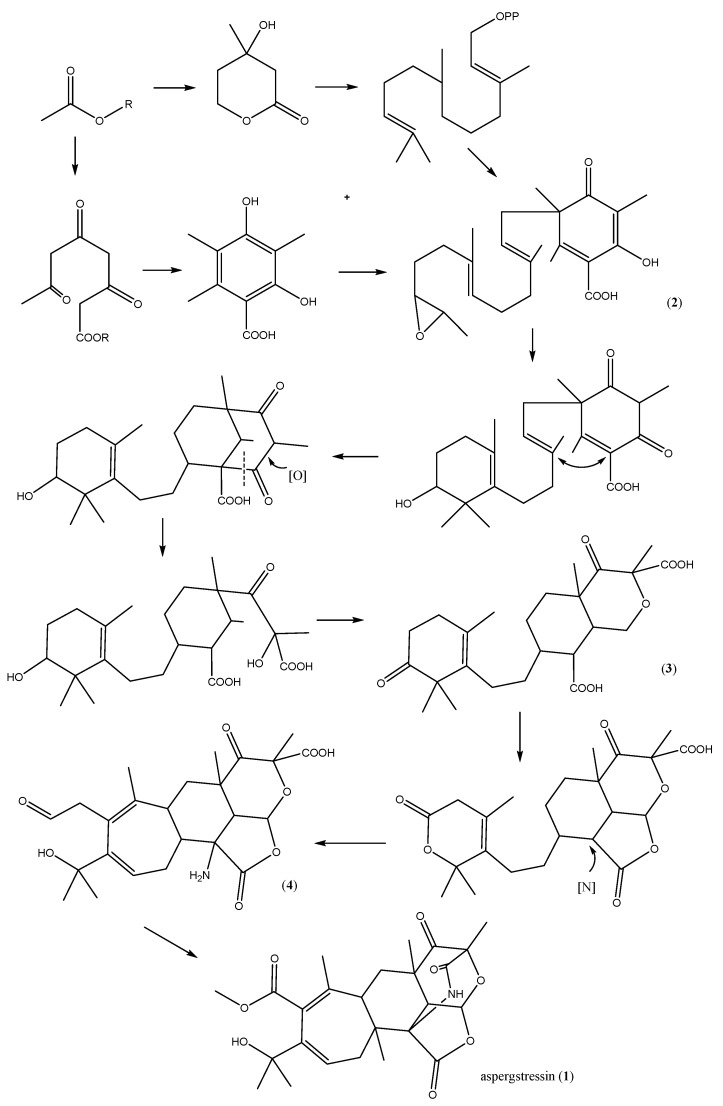
Proposed biosynthetic pathway of compound **1**.

Berkeleyacetals, paraherquonin [14] and citreonigrin [15] are reported polyketide–terpenoids which have similar skeletons to aspergstressin. However, studies on their biosynthesis are scarce. Based on the biosynthetic studies on andibenin A-B [16] and terretonin [17], two other polyketide–terpenoid compounds isolated as *Aspergillus* fungal metabolites, a biosynthetic pathway can be proposed for the new stress-induced metabolite **1** (Scheme 1). Alkylation of 3,5-dimethylorsellinate by the terpenoid precursor farnesyl pyrophosphate gives the important intermediate **2**. Polyketone ring opening and etherification with a hydroxyl forms an epoxy structure by enol tautomerism and oxidation (compound **3**). Amidation at position 7 forms an amide bridge, followed by hydrolysis and rearrangement of the cyclohexene lactone ring to afford aspergstressin (**1**).

## 3. Materials and Methods

### 3.1. General Procedures

The high-performance liquid chromatography (HPLC) system used was composed of a Waters 717 plus Autosampler, a Waters 600 Controller, a Waters 996 Photodiode Array Detector and a Waters Millog workstation (Waters, Shinagawaku, Tokyo, Japan). Optical rotations were recorded on a 341 polarimeter (Perkin-Elmer, Shanghai, China). ^1^H-NMR (600 MHz) and ^13^C-NMR (150 MHz) spectra were measured at 25 °C on an Agilent 600MHz DD2(DirectDrive2) spectrometer (Agilent, Beijing, China) with TMS as internal standard. ESIMS were recorded on a 6460 Triple Quad LC/MS (Agilent, Beijing, China). Prep. HPLC was performed on an Agilent-1100 system equipped with a Venusil MP-C18 column (10 mm) 250 mm (Agela Technologies, Shanghai, China). Sephadex LH-20 (Amersham, Piscataway, NJ, USA) was used for column chromatography. The organic solvents used in chromatographic separation were of analytical grade purchased from Sayfo Technology (Tianjin China) and chromatographic grade for HPLC analysis purchased from Tedia, USA. Deionized water was prepared by Reverse osmosis Milli-Q water (18 MΩ) (Millipore, Bedford, MA, USA) and used for all solutions and dilutions. Agar powder for plate culture and cobalt chloride was purchased from Sinopharm Chemical Reagent Co., Ltd. (Shanghai, China).

### 3.2. Isolation, Cultivation, and Storage of the Strain WU 243

The strain WU 243 was isolated from the crab *Xenograpsus testudinatus*, which was collected from a Kueishantao hydrothermal vent (Taiwan). The strain was grown on PDA agar, consisting of 100 g potato lixivium, 10 g dextrose, 35 g sea salt and 15 g agar powder per litre. The strains were stored in −80 °C cryopreservation box (Forma 700 Freezer, Thermo Scientific, Shanghai, China).

### 3.3. Normal Culture and Metal-Stress Cultivation

Normal cultivation of strain WU 243 was carried out in 500 mL flasks containing 225 mL liquid PDB for 14 days at 24 °C as static cultures in the dark. To investigate the maximum inhibition concentration of cobalt ion and the optimal cultivation conditions, the metal-stress method was first scheduled with four cobalt ion concentrations of 0, 5, 10 and 20 mM, respectively. The mycelium was removed and culture broth was extracted with an equal volume of ethyl acetate. Afterwards, the optimal stress conditions were investigated for more precise ion concentrations from 6 mM to 10 mM. The final ion concentration for stress culture in large scale was determined as 6 mM based on the HPLC profile of each extract and the peak areas of the stress-induced products.

### 3.4. HPLC Analysis and Identification of Stress Metabolites

Analytical reversed phase HPLC-UV experiments were performed using a C_18_ column (sepax Amethyst C18-H, 100 mm × 3.00 mm) applying an H_2_O/methanol (MeOH) gradient from 20% MeOH to 100% MeOH in 30 min, maintaining 100% MeOH for 10 min, flow rate 0.8 mL/min on a LC20A system (Shimadzu, Kyoto, Japan) equipped with a Prominence CBM-20A/20 Alite controller, SPD-20A UV detector, and Prominence CTO-20A column oven. The stress-induced compounds were isolated by preparative HPLC, which was carried out using a HPLC-UV system (P3000 type high pressure infusion pump, UV3000 type ultraviolet/visible light detector, Rheodyne 7725 I manual sampling valve, sepax C18, 150 mm × 80 mm, column). Based on the results on analytical HPLC analysis, a constant mobile phase of 52% MeOH for 30 min and a flow rate of 10 mL/min were employed for isolation and purification of compound **1**.

### 3.5. Confirmation and Fermentation in Large Scale

After defining the optimal cultivation conditions, the strain was cultivated under metal stress conditions in 500 mL of medium in triplicate. The broth was then extracted and the metabolite profiles were checked by HPLC to authenticate the viability of the stress method. Strain WU 243 was inoculated onto agar plates containing PDA medium. After incubation for 7 days at 24 °C, the pre-culture was used for inoculation of two 500 mL flasks containing 225 mL liquid medium (natural pH) as seed bottles. The seed bottles were incubated for 14 days a t 24 °C as static cultures in the dark. This broth was used as seed to amplify the culture scale. Five 5 L flasks which contained 2 L zymotic fluid and one hundred 500 mL flasks containing 200 mL liquid medium were used for large scale fermentation. A total of 20 L of broth containing 6 mM cobalt ions was incubated for 18 days at 28 °C as static cultures.

### 3.6. Extraction and Isolation of Compound ***1***

The 20 L of fermentation broth was extracted with ethyl acetate. After evaporation of the solvent the crude extract was dissolved in methanol. The extract was subjected to preparative HPLC (P3000 type high pressure infusion pump, UV3000 type ultraviolet/visible light detector, Rheodyne 7725 I manual sampling valve, sepax C18, 150 mm × 80 mm, column, flow rate 10 mL/min, UV detector 210 nm), using 52% MeOH as an eluent, to afford aspergstressin (**1**, 2.5 mg, t_R_ = 18.5 min); white powder; ${\left[\alpha \right]}_{D}^{20}$ +274.24 (*c* 0.1, CH_3_OH); UV (MeOH) λ_max_(log ε) 203 (2.13), 227.5 (1.85) nm; ^1^H-NMR and ^13^C-NMR, see Table 1; ESIMS *m*/*z* 474.2[M + H]^+^; HR-TOF-MS *m*/*z* 474.2234 [M + H]^+^ (calcd. For C_25_H_31_NO_8_, 474.2122).

## 4. Conclusions

The distribution of marine fungi is positively correlated to their host distribution [18]. Strain *Aspergillus* sp. WU 243 was isolated from *Xenograpsus testudinatus*, a crab residing around a heavy metal rich hydrothermal vents environment. Despite the fact that previous studies were carried out on the isolation of secondary metabolites from the widely distributed *Aspergillus* spp., as well as its metal adsorption characteristics [19,20,21,22], no studies have focused on the “metal stress” theory, here related to the heavy metal cobalt. Challenging microbes with toxic heavy metals has been proven to be an effective mean to stimulate their cryptic secondary metabolism [5,6,7]. In this study, a novel hybrid polyketide-terpenoid named aspergstressin (**1**), possessing a unique fused polycyclic structure, was induced by cobalt ion stimulation of culture broth of strain *Aspergillus* sp. WU 243. The result show that stress-driven discovery of new natural products from marine fungi is an effective strategy to unveil the untapped reservoir of small molecules produced by species living in the hydrothermal vent environment.

## Supplementary Materials

Supplementary materials can be accessed at: http://www.mdpi.com/1420-3049/21/1/105/s1.
