# Supplementary material for: An Unusual Stress Metabolite from a Hydrothermal Vent Fungus Aspergillus sp. WU 243 Induced by Cobalt
Source: Molecules. 2016 Jan 16;21(1):105. doi: 10.3390/molecules21010105 (PMC6274401; doi:10.3390/molecules21010105)
Supplement: Supplementary file 1 [file molecules-21-00105-s001.pdf]

Supplementary Materials: An Unusual Stress Metabolite Induced by Cobalt from a Hydrothermal Vent Fungus *Aspergillus* sp. WU 243

Chihong Ding, Xiaodan Wu, Bibi Nazia Auckloo, Chen-Tung Arthur Chen, Ying Ye, Kuiwu Wang and Bin Wu

Table of contents

Figure S1. ^{1}H-NMR spectrum for compound **1**.
Figure S2. ^{13}C-NMR spectrum for compound **1**.
Figure S3. Partially enlarged detail of ^{13}C-NMR spectrum for compound **1**.
Figure S4. Partially enlarged detail of ^{13}C-NMR spectrum for compound **1**.
Figure S5. DEPT spectrum for compound **1**.
Figure S6. COSY spectrum for compound **1**.
Figure S7. Partially enlarged detail of COSY spectrum for compound **1**.
Figure S8. HSQC spectrum for compound **1**.
Figure S9. Partially enlarged detail of HSQC spectrum for compound **1**.
Figure S10. Partially enlarged detail of HSQC spectrum for compound **1**.
Figure S11. HMBC spectrum for compound **1**.
Figure S12. Partially enlarged detail of HMBC spectrum for compound **1**.
Figure S13. Partially enlarged detail of HMBC spectrum for compound **1**.
Figure S14. Partially enlarged detail of HMBC spectrum for compound **1**.
Figure S15. Partially enlarged detail of HMBC spectrum for compound **1**.
Figure S16. NOESY spectrum for compound **1**.
Figure S17. Partially enlarged detail of NOESY spectrum for compound **1**.
Figure S18. Partially enlarged detail of NOESY spectrum for compound **1**.
Figure S19. Structure of normal main product ditryptophenaline.
Figure S20. Structure of known compound cyclo-(L-tryptophyl-L-phenylalanyl).
Figure S21. Structure of known compound cordyol C.
Figure S22. Structure of known compound sydonic acid.
Table S1. ^{1}H- and ^{13}C-NMR (CD_3OD-d_4, 500 MHz) data for known compound ditryptophenaline.
Table S2. ^{1}H- and ^{13}C-NMR (CD_3OD-d_4, 500 MHz) data for known compound cyclo-(L-tryptophyl-L-phenylalanyl).
Table S3. ^{1}H- and ^{13}C-NMR (CD_3OD-d_4, 500 MHz) data for known compound cordyol C.
Table S4. ^{1}H- and ^{13}C-NMR (CD_3OD-d_4, 500 MHz) data for known compound sydonic acid.

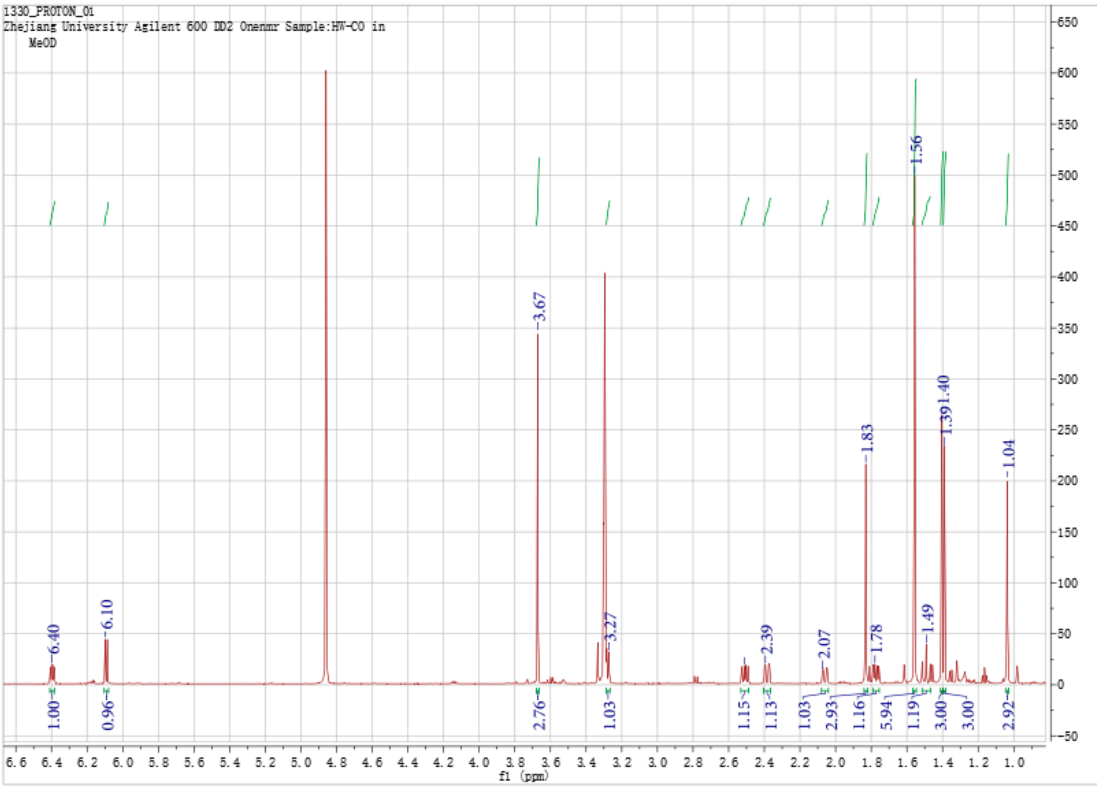

Figure S1. ^{1}H-NMR spectrum for compound **1**.

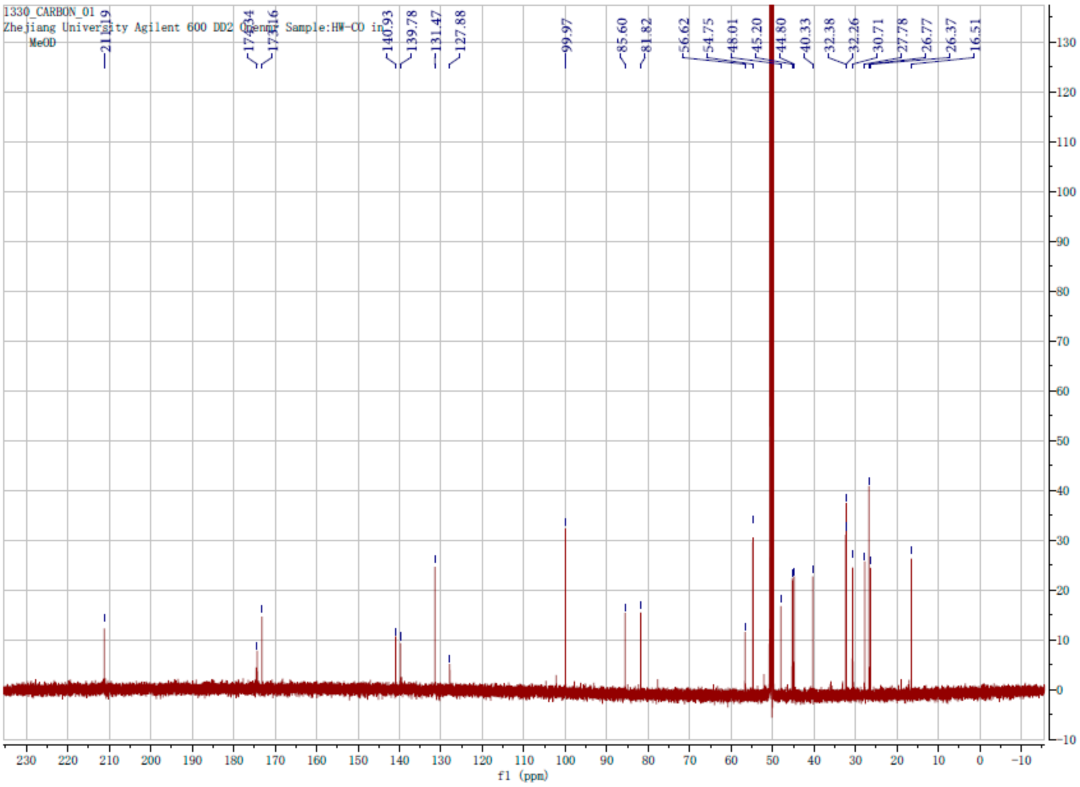

Figure S2. ^{13}C-NMR spectrum for compound **1**.

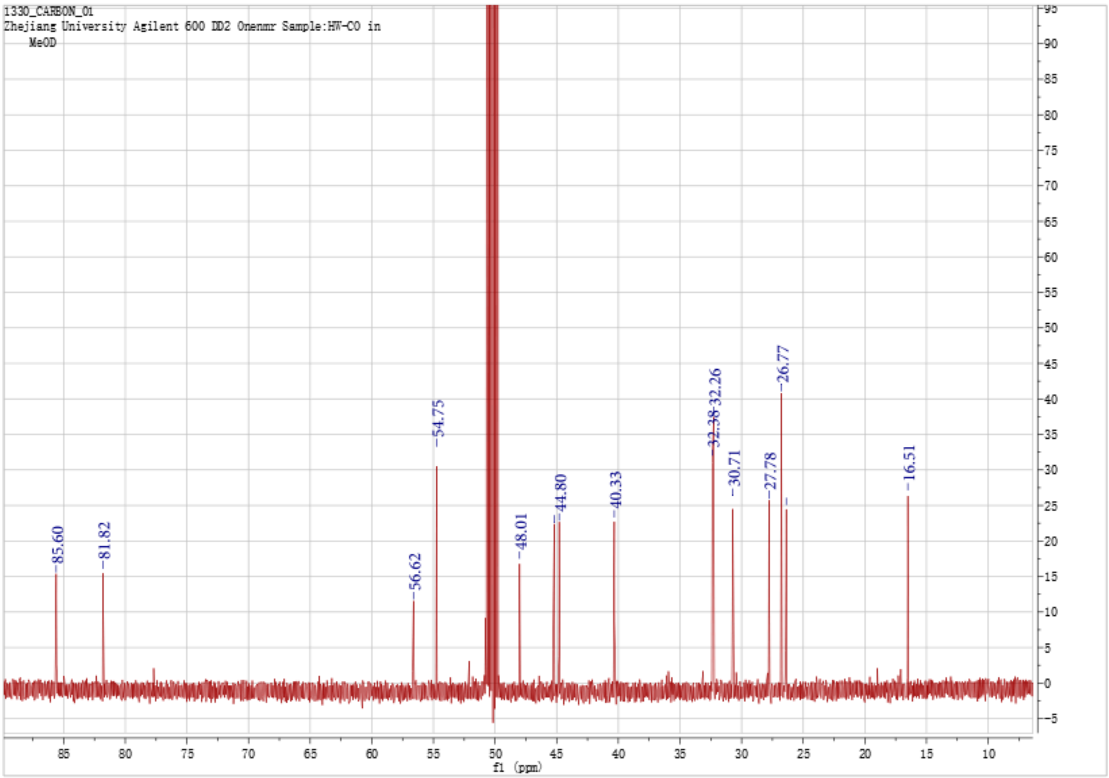

Figure S3. Partially enlarged detail of ^{13}C-NMR spectrum for compound **1**.

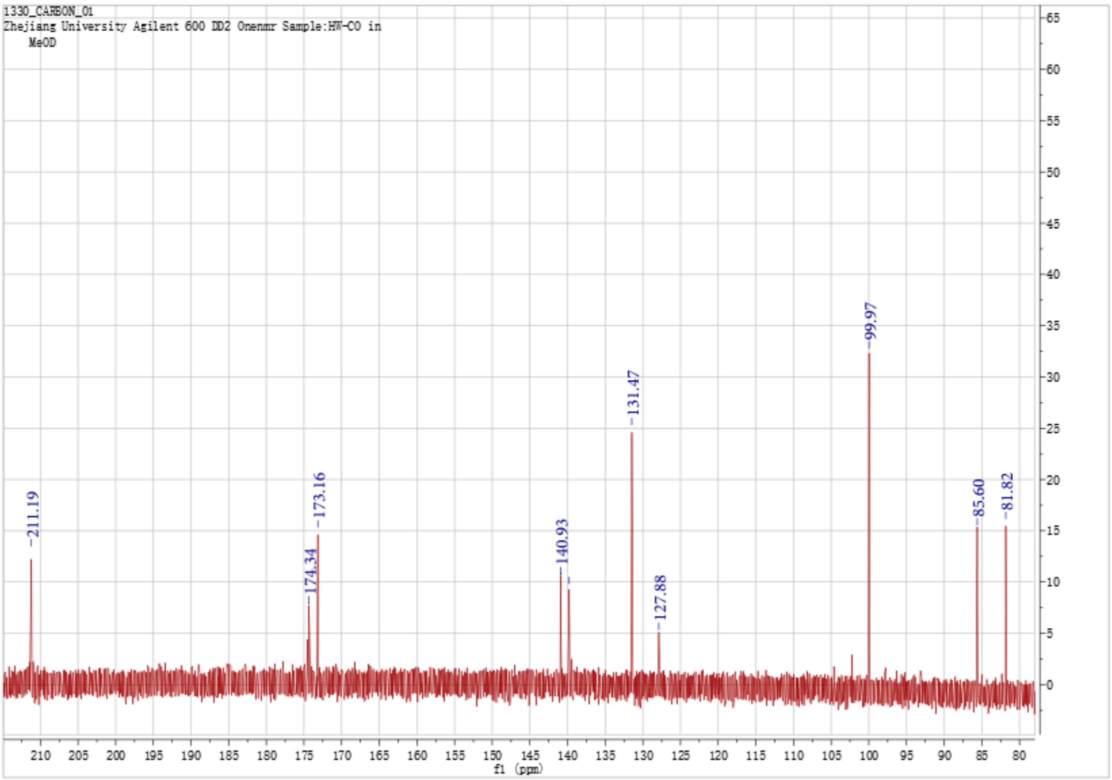

Figure S4. Partially enlarged detail of ^{13}C-NMR spectrum for compound **1**.

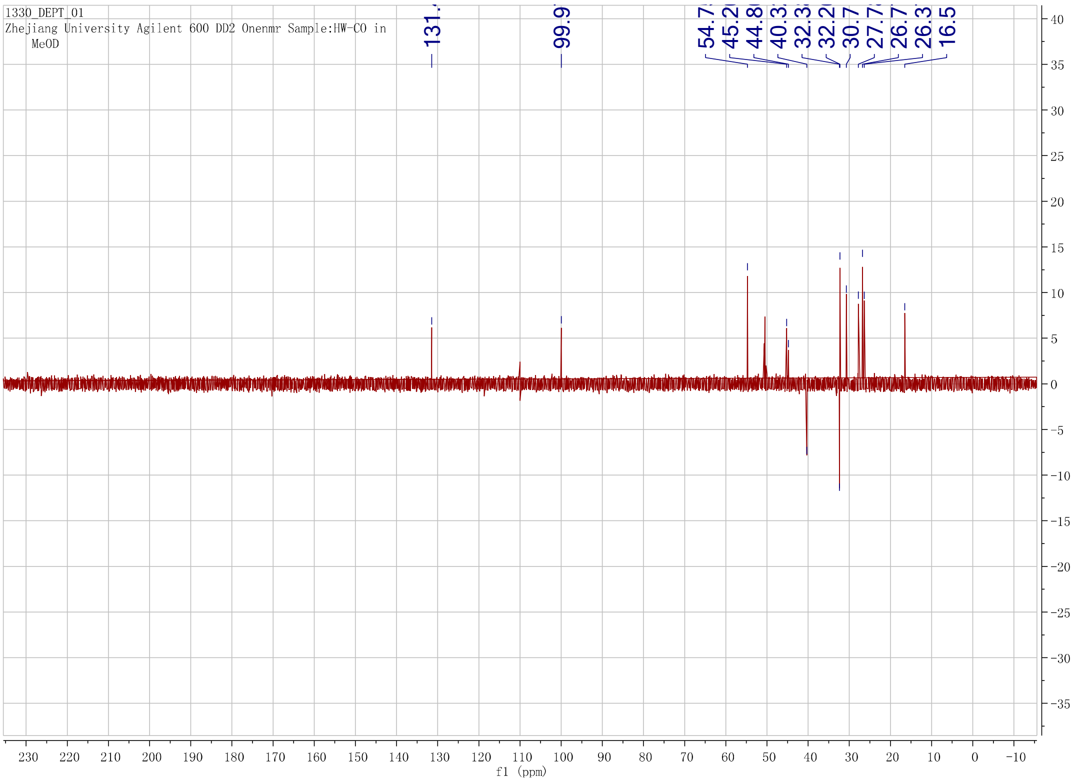

Figure S5. DEPT spectrum for compound **1**.

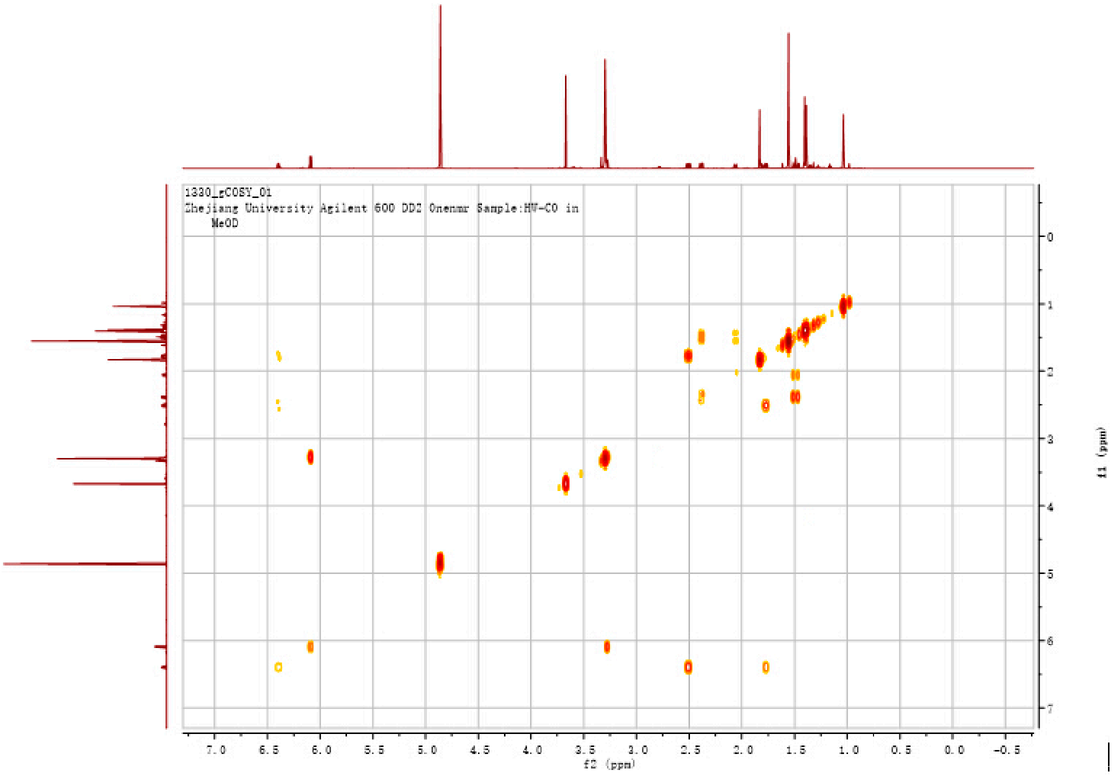

Figure S6. COSY spectrum for compound **1**.

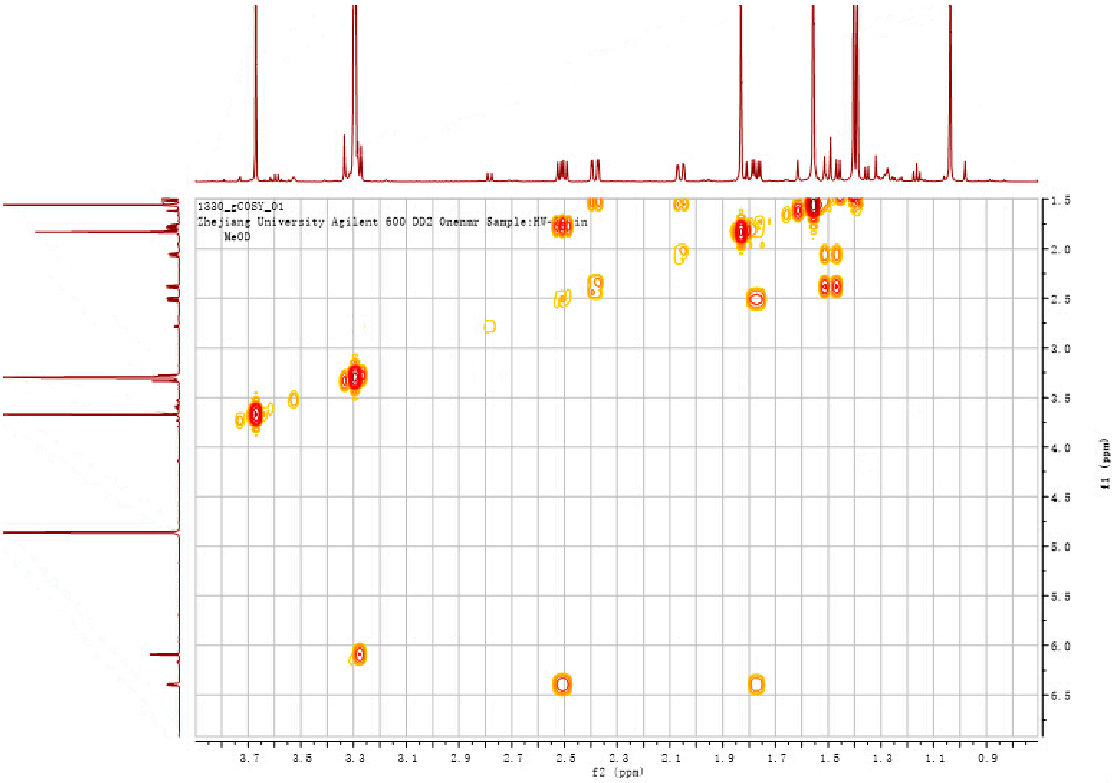

Figure S7. Partially enlarged detail of COSY spectrum for compound **1**.

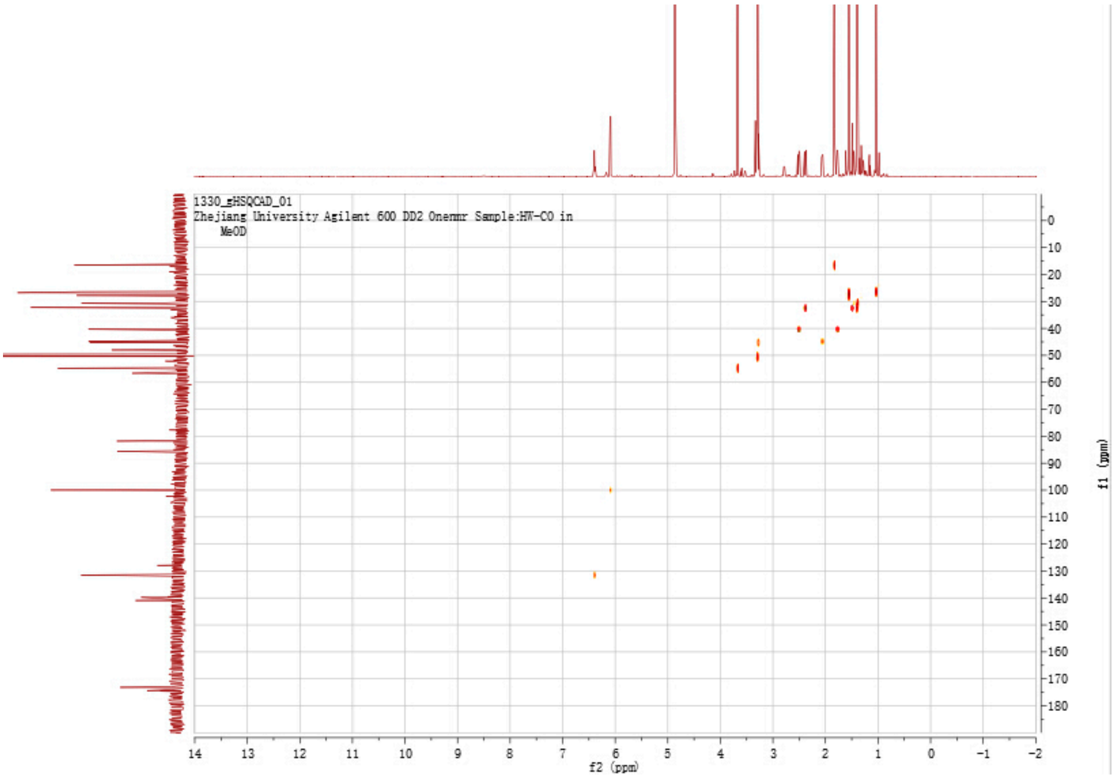

Figure S8. HSQC spectrum for compound **1**.

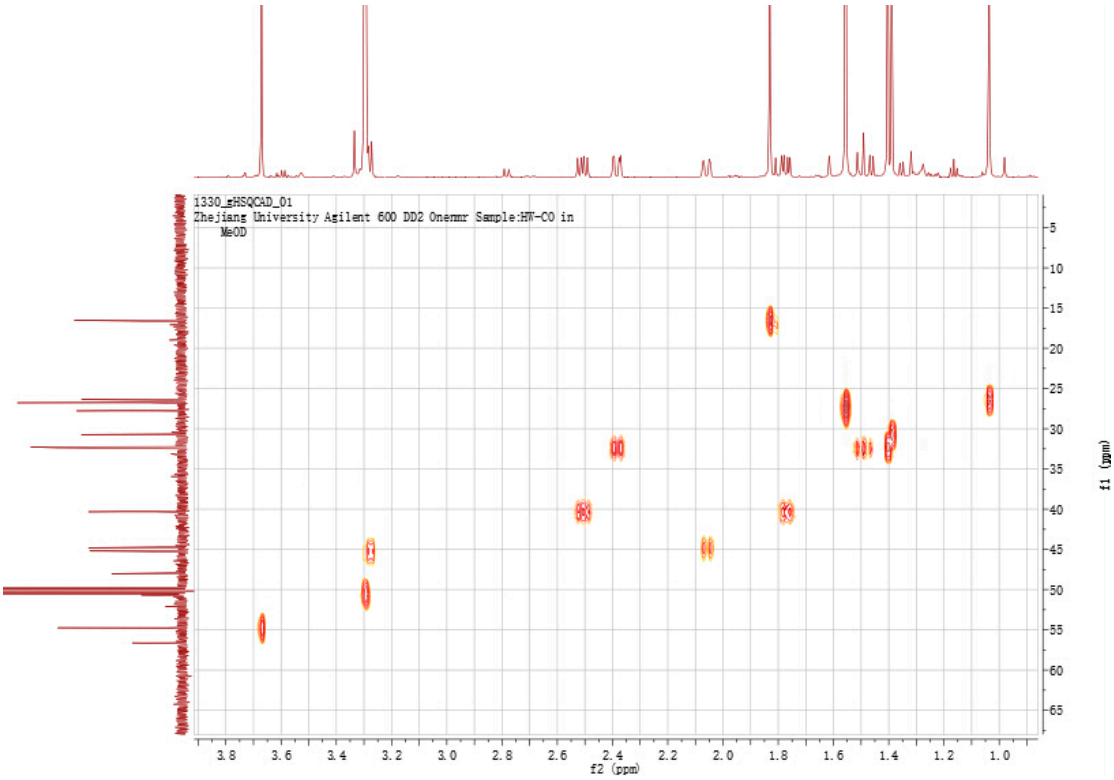

Figure S9. Partially enlarged detail of HSQC spectrum for compound **1**.

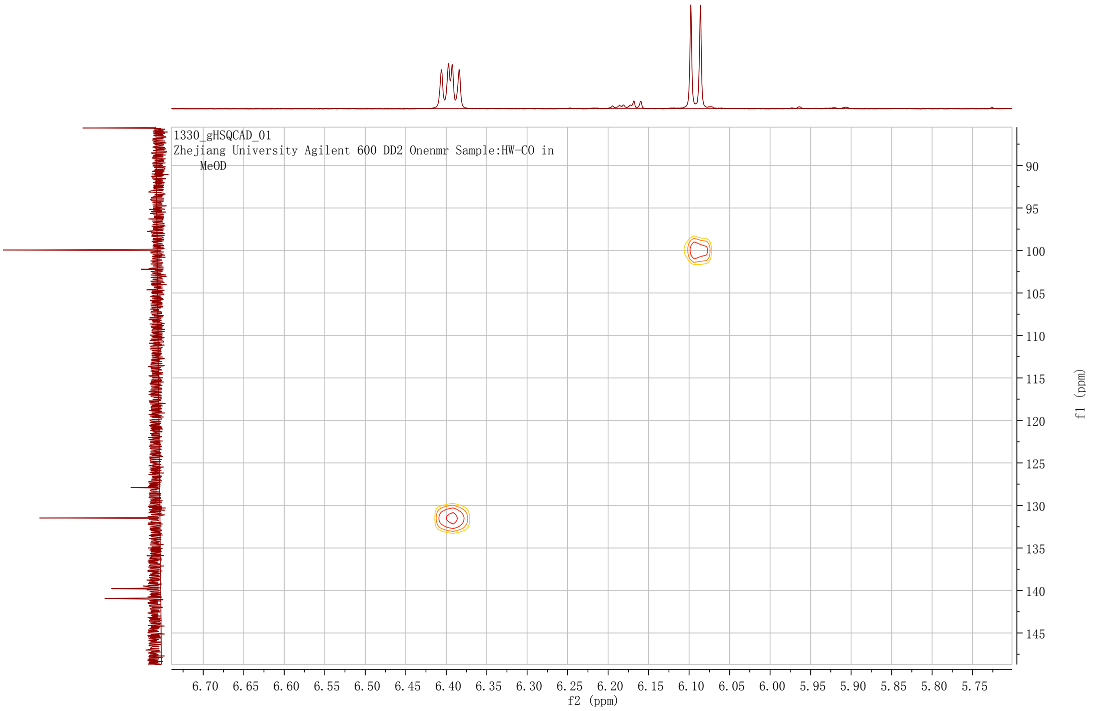

Figure S10. Partially enlarged detail of HSQC spectrum for compound **1**.

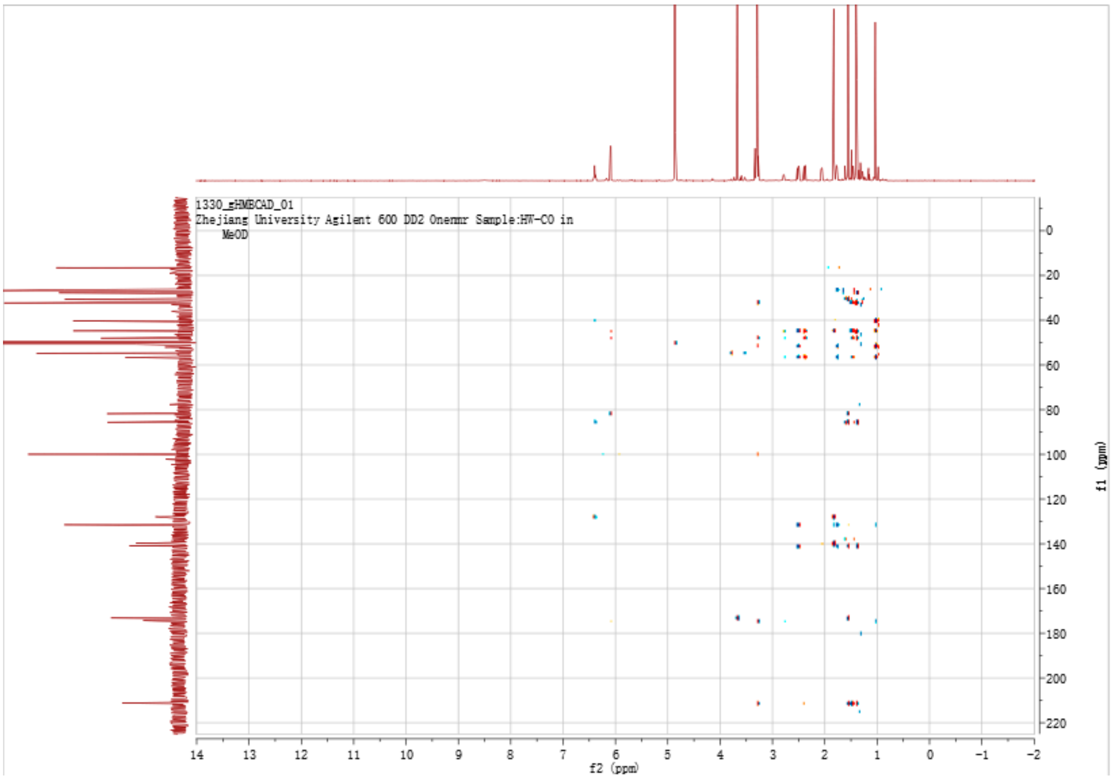

Figure S11. HMBC spectrum for compound **1**.

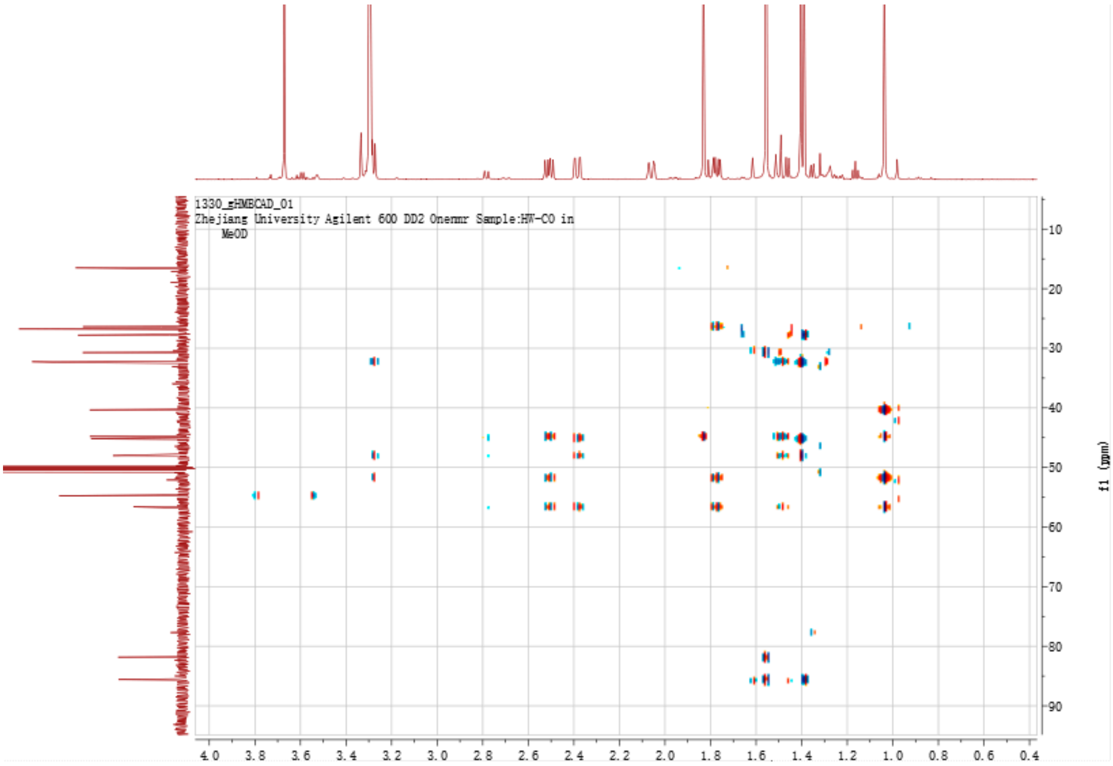

Figure S12. Partially enlarged detail of HMBC spectrum for compound **1**.

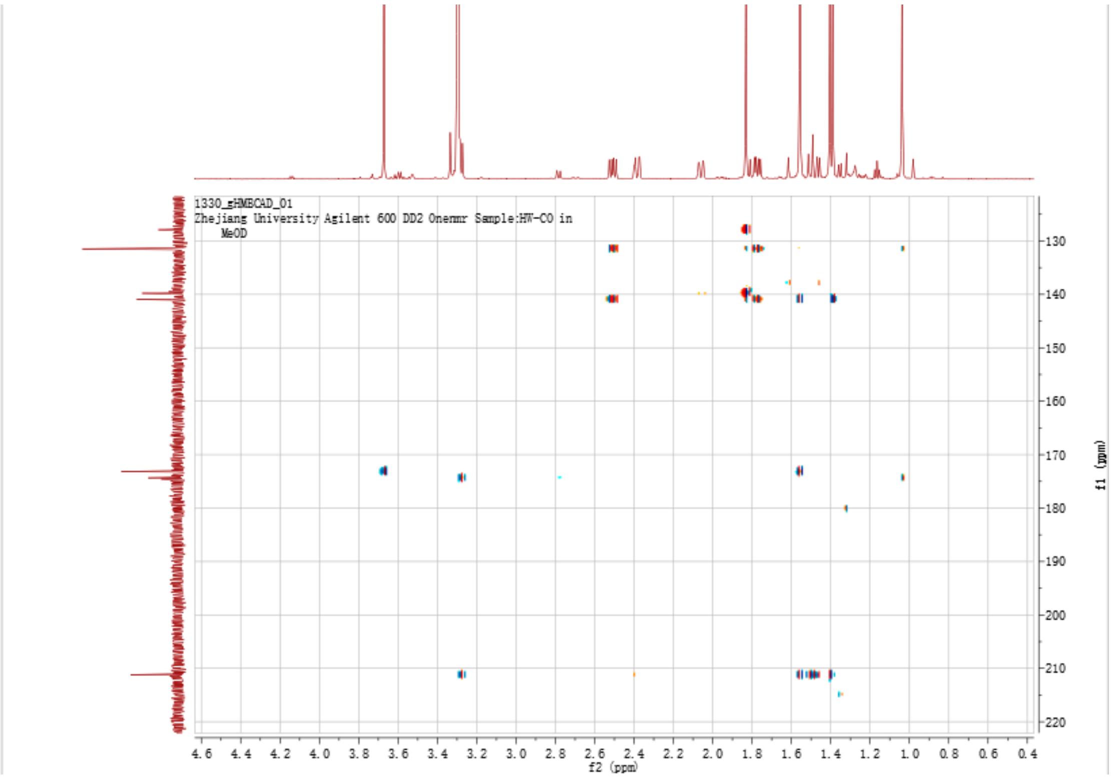

Figure S13. Partially enlarged detail of HMBC spectrum for compound **1**.

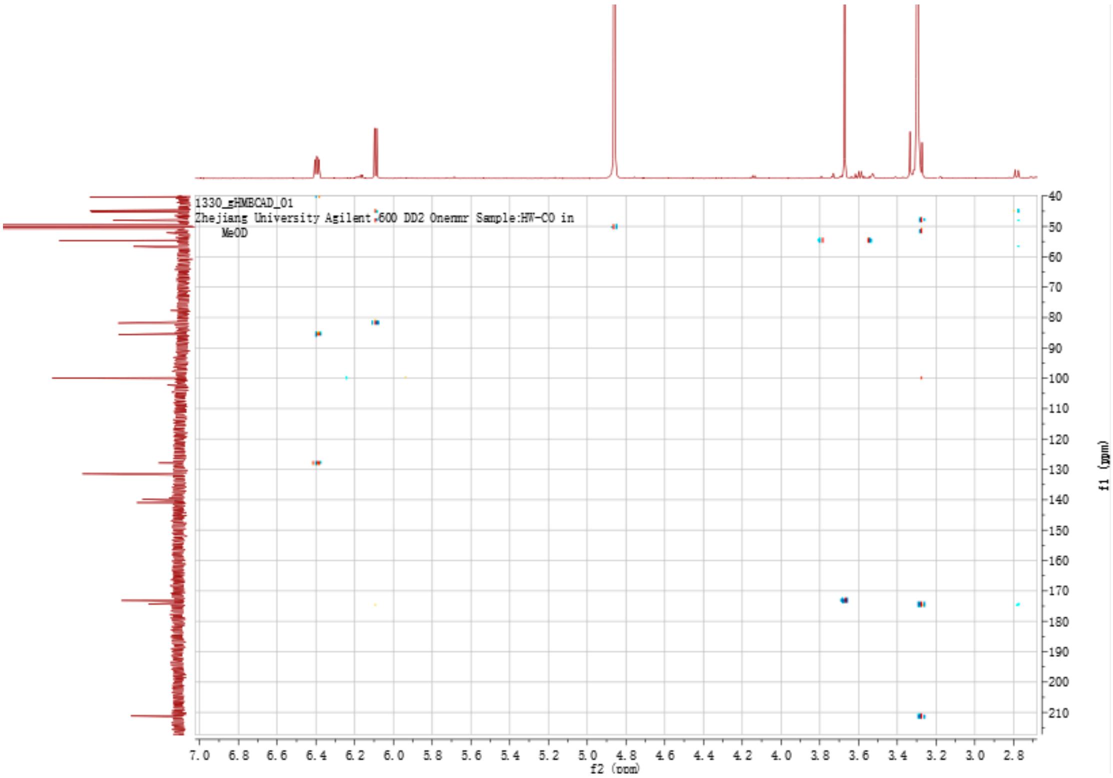

Figure S14. Partially enlarged detail of HMBC spectrum for compound **1**.

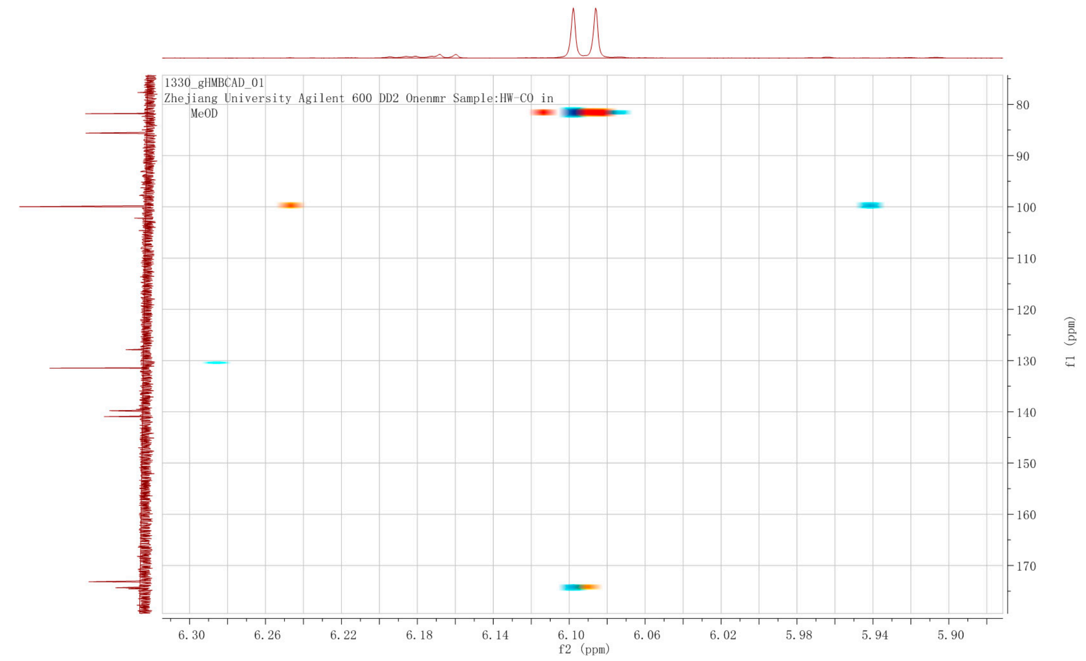

Figure S15. Partially enlarged detail of HMBC spectrum for compound **1**.

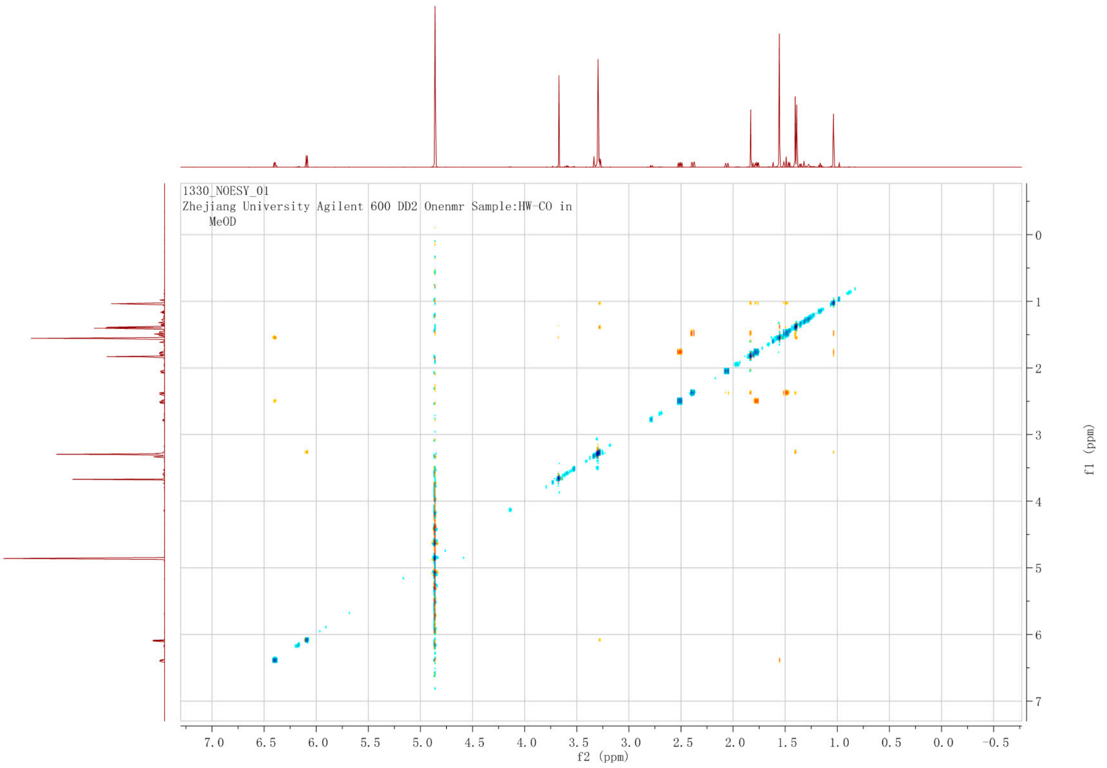

Figure S16. NOESY spectrum for compound **1**.

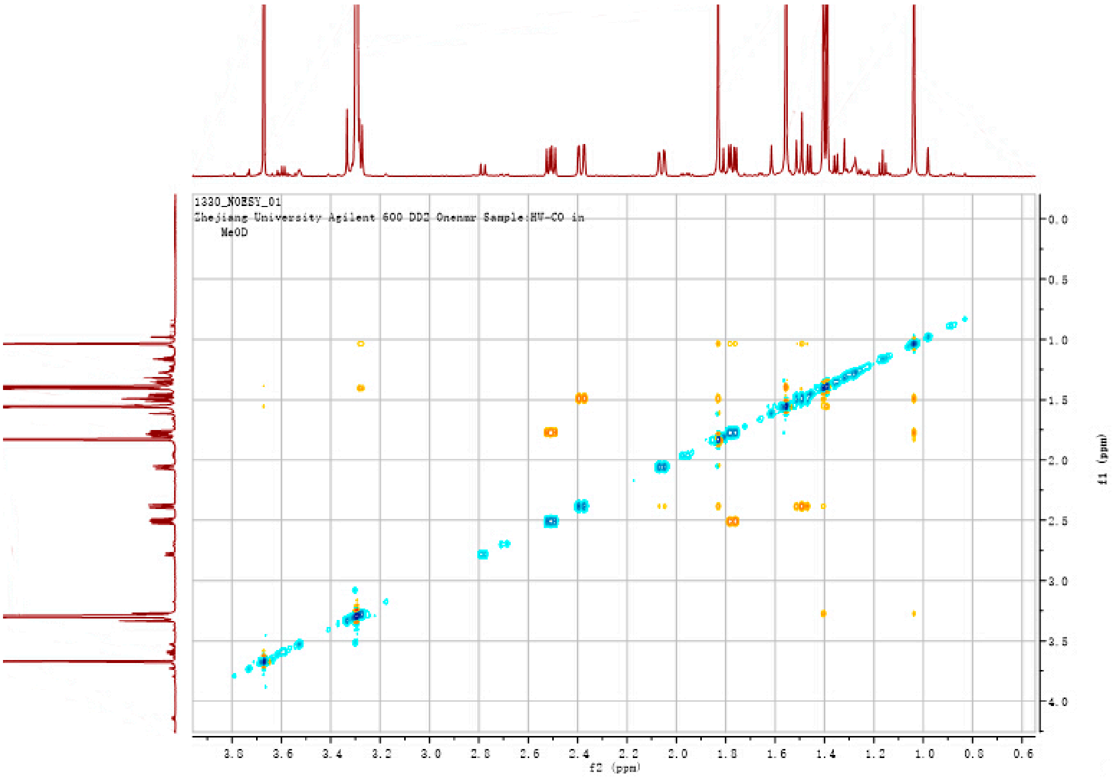

Figure S17. Partially enlarged detail of NOESY spectrum for compound **1**.

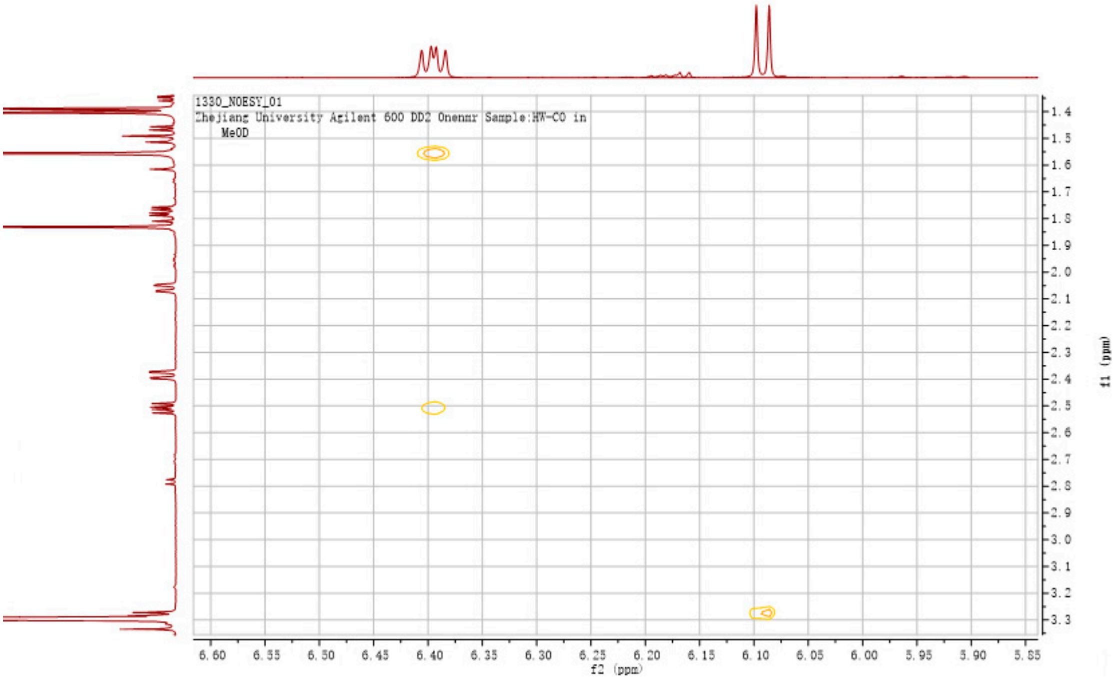

Figure S18. Partially enlarged detail of NOESY spectrum for compound **1**.

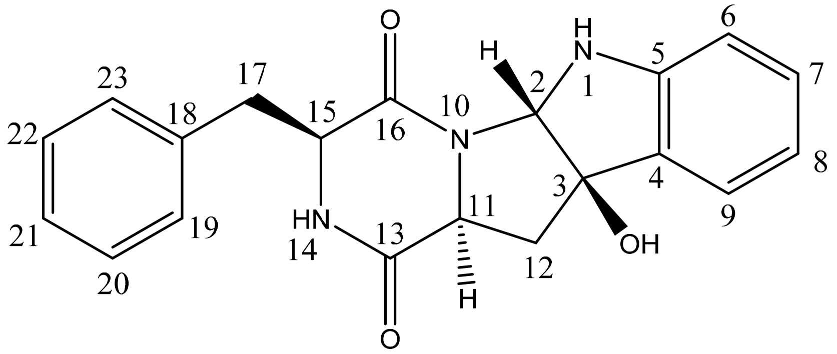

Figure S19. Structure of normal main product ditryptophenaline.

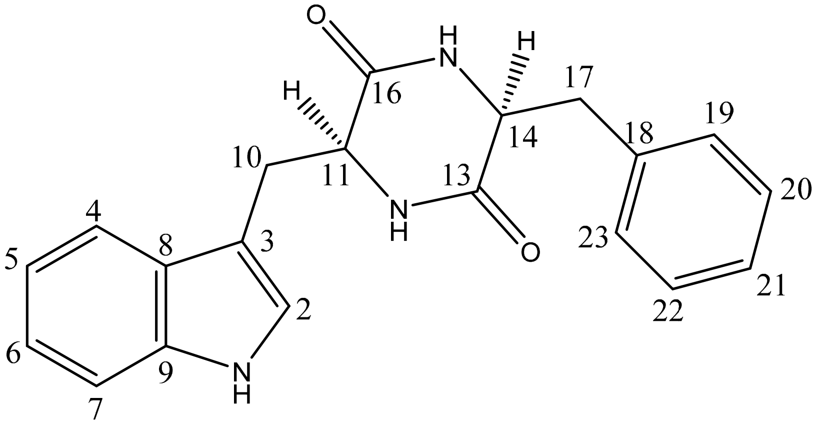

Figure S20. Structure of known compound Cyclo-(L-tryptophyl-L-phenylalanyl).

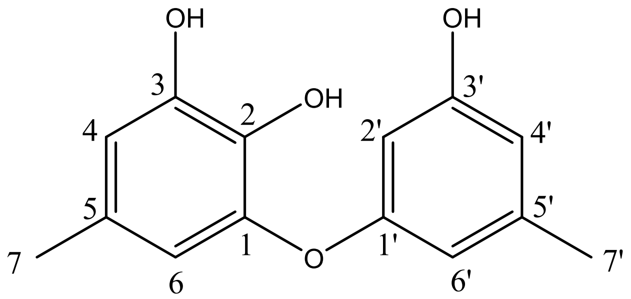

Figure S21. Structure of known compound Cordyol C.

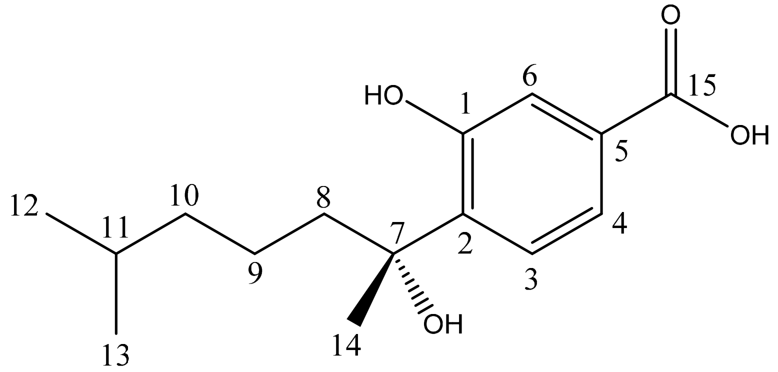

Figure S22. Structure of known compound sydonic acid.

Table S1. ^{1}H- and ^{13}C-NMR (CD_3OD-*d*$_4$, 500 MHz) data for known compound ditryptophenaline.

Position	δH (*J* in Hz)	δC (mult.)
1	N	--
2	4.90(s)	79.45 (CH)
3	--	56.64 (C)
4	--	130.12 (C)
5	--	149.12 (C)
6	6.69 (d,7.8)	109.25 (CH)
7	6.96 (m)	126.23 (CH)
8	6.77 (ddd,0.8,7.4,7.4)	119.02 (CH)
9	7.32 (d,7.4)	124.74 (CH)
10	N	--
11	3.99 (ddd,1.5,8.7,8.7)	56.24 (CH)
12	3.06 (m)<br>2.66 (dd, 1.5,8.7)	36.45 (CH_2)
13	--	169.14 (C)
14	N	--
15	4.19 (ddd,1.6,5,5)	59.70 (CH)
16	--	167.57 (C)
17	3.06 (m)	36.01 (CH_2)
18	--	135.45 (C)
19,23	7.03 (m)	127.86 (CH)
20,22	6.96 (m)	129.06 (CH)
21	7.14 (ddd,0.9,7.8,7.8)	125.15 (CH)

Table S2. ^{1}H- and ^{13}C-NMR (CD_3OD-*d*$_4$, 500 MHz) data for known compound Cyclo-(L-tryptophyl-L-phenylalanyl).

Position	δH (*J* in Hz)	δC (mult.)
1	10.89 (s)	--
2	6.96 (d, 2)	120.83 (CH)
3	--	108.76 (C)
4	7.48 (dd, 8, 8)	118.37 (CH)
5	6.98 (ddd, 8, 8, 1.5)	118.71 (CH)
6	7.07 (ddd, 8, 8, 1)	124.37 (CH)
7	7.32 (dd, 8, 8)	111.29 (CH)
8	1.80 (m)	127.49 (C)
9	1.14 (m)	136.01 (C)
10	2.80 (dd, 14.5, 4.5)	29.66 (CH_2)
11	3.97 (m)	55.23 (CH)
12	7.91 (d, 1.5)	--
13	--	166.13 (C)
14	3.86 (m)	55.58 (CH)
15	7.72 (d, 2)	--
16	--	166.77 (C)
17	1.86 (m)	39.70 (CH_2)
18	--	136.51 (C)
19,23	7.16 (m)	127.99 (CH)
20,22	6.71 (m)	129.66 (CH)
21	7.17 (m)	126.32 (CH)

Table S3. ^{1}H- and ^{13}C-NMR (CD_3OD-d_4, 500 MHz) data for known compound Cordyol C.

Position	δ_H (*J* in Hz)	δ_C (mult)
1	--	145.15 (C)
2	--	135.76 (C)
3	--	147.49 (C)
4	6.45 (d, 1.5)	112.84 (CH)
5		129.87 (C)
6	6.24 (d, 1.5)	113.24 (CH)
7	2.14 (s)	20.70 (CH_3)
1′	--	160.35 (C)
2′	6.16 (dd, 2, 2)	102.56 (CH)
3′	--	159.18 (C)
4′	6.29 (br s)	111.02 (CH)
5′	--	141.14 (C)
6′	6.21 (br s)	110.02 (CH)
7′	2.20 (s)	21.37 (CH_3)

Table S4. ^{1}H- and ^{13}C-NMR (CD_3OD-d_4, 500 MHz) data for known compound Sydonic acid.

Position	δ_H (*J* in Hz)	δ_C (mult.)
1	--	155.94 (C)
2	--	135.36 (C)
3	7.06 (d, 8.0)	126.33 (CH)
4	7.52 (d, 8.0)	121.14 (CH)
5		129.94 (C)
6	7.53 (s)	119.18 (CH)
7	--	78.85 (C)
8	1.80 (m)	42.81 (CH)
9	1.14 (m)	21.62 (CH_2)
10	1.28 (m)	38.99 (CH_2)
11	1.49 (m)	27.75 (CH)
12	0.82 (d, 6.5)	22.52 (CH_3)
13		22.50 (CH_3)
14	1.66 (s)	28.76 (CH_3)
15	--	171.36 (C)
